# Impact of a collaboration revolving around virtual capacity evaluations

**DOI:** 10.1111/1475-6773.14068

**Published:** 2022-09-29

**Authors:** Ronan Factora, Ardeshir Z. Hashmi

**Affiliations:** ^1^ Department of General Internal Medicine Cleveland Clinic Center for Geriatric Medicine Cleveland Ohio USA

**Keywords:** capacity, elder abuse, guardianship, virtual

## Abstract

**Objective:**

To assess the impact of virtual capacity assessments on access to medical care, community supports, and transitions to higher levels of care.

**Study Setting:**

Virtual capacity evaluations of homebound suspected elder abuse/neglect/financial exploitation victims identified via exclusion criteria and initiated by Cuyahoga County adult protective services (APS) and conducted with Cleveland Clinic Geriatric Medicine.

**Study Design:**

A retrospective chart review was conducted in conjunction with APS using their database to determine the outcomes of individuals who underwent virtual capacity evaluation from May 2020 through September 2021. Variables collected included completion of a statement of expert evaluation, guardianship assignment, offering community services, transfer to a higher level of care, and establishment of primary care.

**Data Collection/Extraction:**

Data were extracted from medical records and the APS database. Outcomes were measured as percentages.

**Principal Findings:**

Fifty‐four individuals underwent evaluation. Statements of expert evaluation were completed in 38 cases (70%). Guardianship was assigned in 28 cases (52%). Community services were offered to 51 (89%). Thirty‐one (57%) remained at home. At baseline, only 23 (43%) were receiving primary care. Post evaluation, 44 (81%) were connected or reconnected to their medical provider.

**Conclusion:**

Of individuals who underwent our virtual capacity evaluations, most were able to remain at home, offered community services for support, and linked to primary care.

**What is known on this topic**

- Geriatricians are often asked to assess the decision making capacity of persons where there are concerns about elder abuse/mistreatment
- Limited guidance on how to implement a virtual capacity evaluations program is available in the medical literature
- Little data were published demonstrating the impact that capacity evaluations have on the persons involved

**What this study adds**

- Virtual capacity evaluations can be successfully conducted within the framework of an Age‐Friendly Health System (including the incorporation of cognitive testing (Mentation), medication review/adherence (Medications), gait assessment (Mobility), and working to keep a person at home as much as possible (“What matters most”))
- By collaborating with a community partner, such as adult protective services, the impact of this model on the individuals concerned can be monitored—this includes connection of individuals to appropriate community resources, reestablishment of connection to medical care, and determination of the need for assignment of a guardian
- All evaluation recipients were more likely to be connected to primary care and remain at home even if they were deemed to have impaired decision making capacity but accepted home services.

## 1 INTRODUCTION

Capacity assessments of suspected victims of neglect, abuse, and/or exploitation are often requested of physicians by local adult protective services agencies. Access to such services can be very limited, with one survey conducted in California in 2020 showing 53.1% of counties having no access to such assessments.^1^ Although capacity evaluations are ideally performed in person, virtual assessments can overcome barriers, such as lack of transportation, impairments in ambulation, and refusal by the individual to go to a scheduled physician visit for the evaluation. Virtual capacity evaluation programs have been implemented in various locations in the United States. Guidance on how to implement such programs has been published.^2^ Despite capacity evaluations being performed regularly by clinicians, outcomes of such assessments have been difficult to track, particularly whether or not guardianship was assigned or where persons ended up living after such assessments have been completed. In fact, two surveys conducted looking at guardianship and conservatorship data found that accurate data were lacking at the state levels.^3^, ^4^

Age‐Friendly Health Care Systems highlight four specific elements that represent the foci of evidence‐based quality care for older persons. These include mentation, mobility, medications, and “what matters most” ‐ called the “4Ms.” The virtual capacity evaluation conducted with APS fosters the 4Ms principles in multiple ways.

### 1.1 Mentation

The virtual capacity evaluation predominantly revolves around cognitive function and decision making capacity. Evidence‐based cognitive tests are utilized to assess cognitive function, and decision making capacity is assessed utilizing best practices in determining a person's ability to make medical and financial decisions for themselves.^5^ This data were key in determining if the assignment of guardianship would be recommended following this evaluation.

### 1.2 Mobility

As a component of this evaluation, a virtual assessment of gait (when possible) is conducted as part of the physical examination. Additionally, observations from the adult protective services nurse identify problems with ingress and egress from a person's residence, physical barriers to safe mobility around a person's household, and general observations about a person's walking and balance. This is useful information in assessing the person's safety in the home.

### 1.3 Medications

Additionally, medication reconciliation and pill count are conducted as part of this evaluation in conjunction with the nurse. This also helps provide information regarding a person's comorbidities (especially when access to previous medical records and medical history is limited) and adherence to the medication regimen (based on a pill count from the nurse). Findings from this part of the evaluation inform^1^ as above and help identify potential needs for the individual to maintain them at home and also connect them to medical care if a gap is identified in the management of their chronic illnesses.

### 1.4 Matters most

Maintaining independence and keeping a person in the community through the addition of community resources is a primary goal of this assessment, which is often aligned with the client's wishes and desires, those of the family (in many circumstances), and of the community. Such resources are also helpful in potentially reducing caregiver strain and burden (which can increase the risk of nursing home placement) and may also provide the caregivers with assistance in planning for a move to a higher level of care if needed in the future.

Adult protective services initiate this evaluation once concerns about abuse, exploitation, or neglect are reported, with the goal of identifying the needs of the individual, providing community resources to support them at home if possible, and identifying those that are lacking in medical care. If appropriate, APS also helps those who are unable to remain in the community move to a more supervised setting by fostering realistic discussions about plans of care if that individual was no longer able to stay at home; this helps keep the person involved in the decision making as much as possible, even if cognition is impaired.

Using this as a framework, geriatricians at the Cleveland Clinic implemented a virtual capacity evaluation program in collaboration with Cuyahoga County adult protective services (APS). This was particularly important during the COVID‐19 pandemic when in‐person visits became extremely limited, and the incidence of elder abuse was rising nationally.^6^ The utilization of telehealth visits during the pandemic was shown to be particularly beneficial for older persons.^7^

The need for this evaluation is triggered by APS when cognitive concerns or unaddressed medical issues are identified by the APS social worker who conducts an initial in‐person assessment. Arrangement for another evaluation with an APS nurse is made at that time. The virtual capacity evaluation was conducted in this collaboration with the APS nurse (who is present in person with the patient) and the geriatrician (connected via a video platform remotely to interview the patient.) The APS nurse obtains vitals (including weights), conducts a medical review with pill count, and facilitates the cognitive evaluation conducted by the geriatrician. The presence of the nurse at the patient's home helps overcome potential barriers that an older person may encounter when using newer technologies.^8^ Many of the components and goals of this virtual capacity evaluation integrate the elements of the 4Ms (mentation, mobility, medications, and what matters most).^9^ The geriatrician is able to conduct a limited virtual physical examination that includes an evaluation of gait and mobility. A cognitive evaluation is always attempted; this includes the Saint Louis University Mental Status Examination (SLUMS)^10^ and Trails A and B^11^ (conducted virtually by the geriatrician), as well as the Geriatric Depression Scale (short form),^12^ CLOX 1 and CLOX 2^13^ (implemented by the APS nurse in person). Additional questions asked by the geriatrician focus on the person's memory problems, mood, and function to aid in assessing that person's decision making capacity.^14^

Upon completion of the interview, the geriatrician and the APS nurse connect again privately to discuss observations and findings and develop a plan of care. A statement of expert evaluation (SEE) is completed by the geriatrician in cases where medical and decision making capacity is thought to be impaired. The completed document is provided by APS to Cuyahoga County Probate Court as a medical opinion to assist in determining if a guardian or less restrictive surrogate decision maker is to be assigned.

We sought to determine the impact of our collaboration on the individuals evaluated within the context of the 4Ms.

## 2 METHODS

A retrospective chart review was conducted in conjunction with APS using their database to determine the outcomes of individuals who underwent virtual capacity evaluation from May 2020 through September 2021. Fifty‐four individuals underwent this evaluation.

Data collected included age, gender, whether or not a statement of expert evaluation was completed, if a guardian or conservator was assigned, if community services to support the individual were recommended, if these services were accepted, if the individual was able to remain in their current residence or were placed in a more supervised setting (e.g., nursing home or another long‐term‐care facility), if the individual was receiving routine medical care before this evaluation, if they were connected/reconnected to medical care afterward, if they had a diagnosis of dementia before this evaluation being conducted, and the type or types of elder abuse that were alleged. The data collected were binary in nature (“yes” or “no”) for the variables being studied.

Descriptive statistics were measured to assess the percentage of patients for whom a particular outcome was reached.

## 3 RESULTS

The APS database and the medical records of 54 individuals who underwent a virtual capacity evaluation were reviewed. Table 1 shows the demographic characteristics of this cohort. The average age was 79. Nineteen were men (35%), and 35 were women (65%). Allegations of elder abuse that initiated APS involvement included abuse due to a single cause in 40 cases (74%) and abuse due to multiple causes in 14 cases (26%). Neglect and self‐neglect were the most common single causes of alleged elder abuse, with 30 cases involving this allegation (75%). Financial exploitation was one component in 10 of the cases involving multiple causes of elder abuse (71%).

**TABLE 1:** Demographics and outcomes of clients undergoing virtual capacity evaluation

Statement of expert evaluation completed	Yes	No
	38	16
Men	11	8
Women	27	8
Average age (years)	79.1	78.4
Guardian assigned	28	0
Conservator assigned	3	2
Connected to primary care before evaluation	16	7
Connected to primary care after evaluation	33	11
Dementia diagnosis before evaluation	13	4

Single abuse type alleged	27	13
Neglect (N)	3	0
Self‐neglect (SN)	17	10
Financial exploitation (FE)	7	2
Verbal abuse (VA)	0	1

Multiple abuse types alleged	11	3
Neglect + financial exploitation	3	1
Self‐neglect + financial exploitation	3	2
Physical abuse + financial exploitation	1	0
Neglect + self‐neglect	4	0

Services offered		
Yes	33	15
No	5	1

Services accepted		
Yes	12	3
No	21	12
Stayed home	15	16
Placed into supervised setting	18	0
Other	5	0

A Statement of expert evaluation was completed in 38 cases (70%). For these individuals, completion of the SEE reflected a clinical determination that there was impairment in their medical and financial decision making capacity and that assignment of a surrogate decision maker (in the form of a guardian) might be appropriate. Of these 38 individuals, only 13 (38%) had a diagnosis of dementia before this evaluation. A guardian was assigned by the Probate Court of Cuyahoga County in only 28 (74%) of these cases, and a conservator in three of these cases. Community services to support these persons were offered by APS to 48 of the 54 cases (89%) and refused by 33 patients (61%). Before undergoing this evaluation, 23 of 54 persons (43%) were already connected to routine medical care, but afterward, with the assistance of APS, an additional 21 individuals were newly connected to routine medical care, resulting in 44 of 54 persons (81%) having access to primary care after the evaluation was completed.

All 16 individuals who were deemed to have intact decision making capacity (no SEE completed) were able to stay at home regardless of whether or not they accepted community services to support them. For the remaining 38 that were thought to have impaired decision making capacity, 15 remained home (39%), 18 (47%) were moved to a more supervised setting (e.g., nursing home/long‐term care [LTC]), and five (13%) moved in with other caregivers (e.g., family).

Table 2 details the final location of residence determined before the closure of the APS case based on whether they accepted community services that were offered or not. Of the 38 individuals for which SEE was completed, 33 (87%) were offered services. Twenty‐one of 33 refused services (64%), of which 13 (62%) were placed in a more supervised setting, seven (33%) remained home, and one moved in with other family members. Comparatively, of the 12 persons who accepted services, seven (58%) remained home, four (33%) were placed in a more supervised setting, and one moved in with other family members.

**TABLE 2:** Final locations of residence

	SEE completed		No SEE completed		Guardian assigned		No guardian assigned	
Services accepted	Yes	No	Yes	No	Yes	No	Yes	No
	12	21	3	12	9	18	3	3
Stayed home	7	7	3	12	5	5	2	2
Placed	4	13	0	0	4	12	0	1

*Note*: A guardian was assigned only if a statement of expert evaluation (SEE) was completed.

Of the 28 individuals who were eventually assigned a guardian by the court, 27 were offered services, of which 9 (33%) accepted and 18 (37%) refused. Of these nine persons who accepted services, five (56%) stayed at home, and four (44%) were placed in a more supervised setting. Comparatively, of the 18 persons who refused services, five (28%) stayed at home, and 12 (67%) were placed into LTC. The remaining one person moved in with another family member.

Of the 10 individuals who were not assigned a guardian by the court, six were offered services of which three (50%) accepted and three (50%) refused. Of those who accepted services, two remained at home, one moved in with another family member. Of those who refused services, two stayed at home and one was placed into LTC.

An analysis was conducted to look at the relationship between the outcomes of this assessment process and the previous connectedness of the client to a primary care provider—this data are detailed in Table 3. Of the 54 persons who underwent this evaluation, 23 (43%) were connected to a primary care provider prior to the evaluation, whereas 31 (57%) were not. Comparing clients who were connected to primary care prior to the evaluation to those who were not, 16 (70%) versus 22 (21%) had a SEE completed, 11 (48%) versus 17 (55%) had a guardian assigned, 16 (70%) versus 15 (48%) remained at home, and five (22%) versus 13 (42%) were placed into LTC.

**TABLE 3:** Outcomes of clients evaluated based on previous connection to primary care provider

	Connected to primary care before evaluation	No primary care before evaluation
	23	31
SEE completed	16	22
Guardian assigned	11	17
Stayed at home	16	15
Placement	5	13

Abbreviation: SEE, statement of expert evaluation.

## 4 DISCUSSION

Involvement of APS is often accompanied by assumptions that the concerned individual is likely to be assigned a guardian and placed in a nursing home—this is a common misconception.^15^ Resources and community services were offered in a vast majority of the cases in our study (48 of 54 cases)—this is consistent with current practice within the State of Ohio.^16^ Regardless of whether a person was assigned a guardian or not, the probability of being placed in LTC was similar for those who refused or accepted community services. For the 16 persons who did not have a SEE completed (and no guardian was assigned), all remained at home whether they accepted services or not. Yet for those who were assigned a guardian (28 persons), a higher percentage of these individuals who accepted community services were able to stay at home compared to those who did not: 5 of 9 (56%) versus 5 of 18 (28%) as noted in Table 2. Community services appear to be helpful in keeping persons living in their own homes by serving as an intervention to help support these persons. They also seem to be key in preventing LTC placement. These interventions and outcomes would seem to contradict this misconception of APS's role in the evaluation and management of the cases they encounter.

Our study's findings are in‐line with other published data regarding the percentage of individuals who were found to have impaired capacity, but a greater fraction of individuals in our study appear to have been assigned a guardian. For 38 of 54 (70%) individuals who underwent this evaluation, a SEE was completed, with 28 (52%) eventually being assigned a guardian. In another published study conducted in Texas,^17^ 68% of individuals undergoing a mental health assessment were determined to lack capacity, with the need for guardianship determined in 45 of 216 cases—the authors of that study reported that court‐appointed guardianship findings were not available at the time of publication. This difference in the assignment of a guardian could reflect differences in utility and availability of other solutions available at each study's location to address the problems encountered. Successful assignment of a conservator, payee to help manage finances, or involvement of family members and community agencies to support the individual involved may have been viable alternatives that were accessed.

Persons who underwent this evaluation also appeared to lack routine medical care. The APS nurse obtained vitals and performed a detailed medication review (also assessing adherence and confirming potential medical comorbidities), and the geriatrician performed a limited virtual physical examination along with the cognitive evaluation. Identification of poorly managed comorbidities through a review of available medical records helped to highlight medical issues that may threaten that person's health and independence. Only 17 of 54 people in this study (31%) had a diagnosis of dementia before the completion of the assessment. After completion of the assessment, Table 1 shows that a large portion of persons assessed were either newly connected (21 persons) or reconnected (23 persons) to medical care; 81% of persons were connected to primary care at the end of this evaluation compared to just 43% beforehand. Re‐establishing access to primary care should increase the probability of managing these comorbidities, diagnosing dementia, and mitigating any potential threat to a person's independence.

Our study has several limitations. The results of our analysis likely reflect the practice pattern and type of clients encountered within Cuyahoga County. There is also limited published data regarding the outcomes of virtual capacity evaluations performed in other parts of the country,^3^, ^4^ but our study was able to provide some insight into the outcomes that may occur. Our cohort is small. Selection criteria for persons for which virtual capacity evaluation is conducted likely reflect the practice in Cuyahoga County. Persons who undergo virtual capacity evaluations likely represent a unique subset of individuals traditionally referred to APS for evaluation, and outcomes of their cases may not be representative of the typical case as it advances through an APS investigation. Comparative analysis of these outcomes remains challenging as the data on the outcomes of APS cases within the state of Ohio, and as a whole, nationally is of limited availability.

The literature on virtual capacity evaluations is extremely limited, but the implementation of such evaluations can provide benefits to those who cannot be evaluated through traditional means. The virtual capacity program that we implemented with APS achieved many goals espoused within the 4Ms. We successfully addressed the “Mentation” component in our evaluation by assessing the client's cognition and decision making. We were able to address the “Mobility” component by conducting an evaluation of gait within and an environmental assessment of fall risk within the context of a person's residence. We were successful in addressing the “Medications” component of the 4Ms by connecting a large fraction of persons to a primary care provider to ensure appropriate management of the medical comorbidities. Additionally, we showed that persons who are evaluated and determined to lack decision making capacity, have a guardian assigned, and are offered and accept community resources are more likely to remain at home than those who refuse—helping to achieve “what matters most” for this group of individuals.

Ultimately, our data show that geriatric medicine collaboration with adult protective services, even through the use of a virtual platform, positively impacts socially isolated persons who have experienced one or multiple forms of elder mistreatment or neglect by connecting them to essential community services to keep them at home, facilitating arrangement of appropriate surrogate decision makers, reconnecting them to needed medical care, and successfully keeping many of them out of long‐term‐care facilities—all outcomes that reflect the focus of the 4Ms. Although larger studies will certainly be helpful in showing the impact that this sort of program can have in a community, we hope our work can be used to foster continued dissemination of this innovative approach to care and help other institutions implement a similar program.

## FUNDING INFORMATION

There was no funding provided for this study.
